# New insights into the genus *Gyroporus* (Gyroporaceae, Boletales), with establishment of four new sections and description of five new species from China

**DOI:** 10.1080/21501203.2022.2094012

**Published:** 2022-07-05

**Authors:** Ming Zhang, De-Chun Xie, Chao-Qun Wang, Wang Qiu Deng, Tai-Hui Li

**Affiliations:** a State Key Laboratory of Applied Microbiology Southern China, Guangdong Provincial Key Laboratory of Microbial Culture Collection and Application, Institute of Microbiology, Guangdong Academy of Sciences, Guangzhou, Guangdong Province, China; b Department of Public Health Laboratory Sciences, School of Public Health, Hengyang Medical School, University of South China, Hengyang, Hunan, China

**Keywords:** Eastern Asia, new taxa, phylogenetic analysis, taxonomy

## Abstract

Species of *Gyroporus* from southern China were studied in this study. Based on morphology and molecular phylogenetic analyses of DNA sequences from the nuclear ribosomal internal transcribed spacer (ITS), the nuclear ribosomal large subunit (nrLSU), and the mitochondrial adenosine triphosphate ATP synthase subunit 6 (*atp6), Gyroporus* was divided into four main branches in the phylogenetic tree, and four sections were firstly proposed i.e. *Gyroporus* sect. *Castaneus, G*. sect. *Cyanescens, G*. sect. *Longicystidiatus* and *G*. sect. *Pallidus*. Five new species, i.e. *G. alboluteus, G. atrocyanescens, G. pseudolongicystidiatus, G. pallidus* and *G. subcaerulescens*, were revealed from China, and their phylogenetic positions were also analysed. Among them, *G. alboluteus* and *G. pallidus* were nested into the sect. *Pallidus*, although morphologically similar to *G. castaneus; G. atrocyanescens* and *G. subcaerulescens*, with obvious cyanescent oxidation reactions, were nested into the sect. *Cyanescens*; and *G. pseudolongicystidiatus* characterised by its long cystidia and was nested into the sect. *Longicystidiatus*. The new species were formally described and illustrated in the present study, and a key to the sections and species of *Gyroporus* in China was provided.

## Introduction

*Gyroporus* Quél., typified by *G. cyanescens* (Bull.) Quél., is a small but poorly understood bolete genus in the family Gyroporaceae of Boletales. Members of *Gyroporus* are widely scattered throughout temperate, subtropical and tropical regions of the world, and strongly implicated as symbionts with an array of ectotrophic plants, such as Fabaceae, Fagaceae, Myrtaceae, Pinaceae, Phyllanthaceae, etc. (Singer et al. 1983; Agerer 1999; Raidl et al. 2006; Watling 2006, 2008; Wilson et al. 2012). Species in *Gyroporus* can be easily identified by the brittle and hollow stipe, the white to yellowish white hymenophore unchanging or changing to blue when bruised, white spore print, ellipsoid basidiospores and the presence of clamp connections (Singer 1986; Watling 2008; Das et al. 2017; Magnago et al. 2018; Huang et al. 2021; Xie et al. 2022). However, it is extremely complicated to determine their taxonomic positions at the species level owing to the overlap of phenotypic variation among species. Recently, molecular phylogenetic studies have provided more effective and accurate evidences for species identification of *Gyroporus*, and some new species have been reported (Das et al. 2017; Magnago et al. 2018; Huang et al. 2021; Xie et al. 2022), while the gene of mitochondrial adenosine triphosphate ATP synthase subunit 6 (*atp6*) has been identified as a utility DNA barcoding marker to determine the infrageneric relationships of *Gyroporus* (Davoodian et al. 2018; Huang et al. 2021).

In China, sixteen species have been recorded, including eight species originally reported from China, i.e. *G. alpinus* Yan C. Li, C. Huang & Zhu L. Yang, *G. brunneofloccosus* T.H. Li, W.Q. Deng & B. Song, *G. flavocyanescens* Yan C. Li, C. Huang & Zhu L. Yang, *G. memnonius* N.K. Zeng, H.J. Xie & M.S. Su, *G. porphyreus* N.K. Zeng, H.J. Xie & Zhi Q. Liang, *G. pseudomicrosporus* M. Zang, *G. subglobosus* N.K. Zeng, H.J. Xie, L.P. Tang & M. Mu, and *G. tuberculatosporus* M. Zang (Zang 1986; Zang et al. 1996; Li and Song 2003; Huang et al. 2021; Xie et al. 2022).

However, a recent study proved that *G. pseudomicrosporus* is a member of *Gyrodon* Opat. (Huang et al. 2021). Although *G. castaneus* (Bull.) Quél. and *G. cyanescens*, originally reported from Europe, have been widely reported in China (Chiu 1948, 1957; Zang 1986; Bi et al. 1994; Wu et al. 2013; Tang 2015; Xie et al. 2022), recent phylogenetic studies indicated that *G. castaneus* might be only distributed in northeastern China; and there were no conclusive specimens or molecular data to prove the natural distribution of *G. cyanescens* in China (Huang et al. 2021), instead, four similar species of *G. memnonius, G. paramjitii* K. Das, D. Chakraborty & Vizzini, *G. porphyreus* and *G. subglobosus* were identified from subtropical and tropical regions of China (Xie et al. 2022).

In recent years, some collections of *Gyroporus* were found in southern China, further study based on both morphological data and molecular sequences from the nuclear ribosomal internal transcribed spacer (ITS), the nuclear ribosomal large subunit (nrLSU) and the gene of *atp6* proved that they represent five species new to science; and phylogenetic analyses using the molecular data from all species with known sequences worldwide revealed that the genus could be divided into four new sections. The result should contribute to further understanding the species diversity of *Gyroporus* in China and the taxonomic relationships of the infrageneric taxa.

## Materials and methods

### Morphological studies

Photographs of the fresh basidiomata were taken in the field. Specimens were dried and deposited in the Fungarium of Guangdong Institute of Microbiology (GDGM). Descriptions of macro-morphological characters and habitats were obtained from photographs and field notes. Colour codes followed Kornerup and Wanscher (1981). Microscopic observations were carried out on tissue sections stained with 5% KOH and 1% aqueous Congo red under a light microscope (Olympus BX51, Tokyo) with magnification up to 1000 × . All measurements were made in 5% KOH. For basidiospore descriptions, the notation (a–)b–c(–d) describes basidiospore dimensions, where the range b–c represented 90% or more of the measured values and “a” and “d” were the extreme values; Q referred to the length/width ratio of an individual basidiospore and Q_m_ referred to the average Q value of all basidiospores ± sample standard deviation. All line-drawings of microstructures were made based on rehydrated materials.

### DNA extraction, PCR amplification and sequencing

Genomic DNA was extracted from the voucher specimens using the Sangon Fungus Genomic DNA Extraction kit (Sangon Biotech Co. Ltd., Shanghai, China), according to the manufacturer’s instructions. Primer pairs ITS5/ITS4 (White et al. 1990), LR0R/LR5 (Vilgalys and Hester 1990), and atp6-2/atp6-3 (Kretzer and Bruns 1999) were used for amplifying ITS, nrLSU and *atp6*, respectively. PCR reactions was performed in a total volume of 25 μl containing 0.5 μl template DNA, 11 μl distilled water, 0.5 μl of each primer and 12.5 μl 2 × PCR mix (DreamTaq^tm^ Green PCR Master Mix, Fermentas). Amplification reactions were performed in a Tprofessional Standard Thermocycler (Biometra, Göttingen, Germany) under the following conditions: 95°C for 4 min; then 35 cycles of denaturation at 94°C for 60s, annealing at 53°C for 60s, and extension at 72°C for 60s; with a final extension at 72°C for 8 min. The PCR products were electrophoresed on 1% agarose gels and sequencing was performed on an ABI Prism® 3730 Genetic Analyser (PE Applied Biosystems, Foster, CA, USA) at the Beijing Genomic Institute (BGI) using the same PCR primers. The raw sequences were assembled and checked with SeqMan implemented in Lasergene v7.1 (DNASTAR Inc., USA). The newly generated sequences in this study were submitted to GenBank.

### Phylogenetic analyses

Sequences generated in this study and those downloaded from GenBank were combined and used for phylogenetic reconstruction. Detailed information of specimens included in this study was given in Table 1. Sequence matrix of ITS, nrLSU and *atp6* were separately aligned with software MAFFT v7 using the E-INS-i strategy (Katoh and Standley 2013) and manually adjusted in MEGA 6 (Tamura et al. 2013).

**Table 1.** Information on specimen used in phylogenetic analyses. Sequences newly generated in this study are indicated in bold.

Taxa	Voucher	Locality	GenBank accession number			References
			ITS	nrLSU	*atp6*	
*G. alboluteus*	GDGM25474-1	China	-	ON502925	ON087643	This study
*G. alboluteus*	GDGM25474-2	China	-	-	ON087644	This study
*G. alboluteus*	GDGM86706	China	ON502903	ON502926	ON087645	This study
*G. allocyanescens*	REH9700A	Queensland	-	-	MF818179	Davoodian et al. 2018
*G. alpinus*	Li1478a	China	MW149438	MW151268	MW452609	Huang et al. 2021
*G. alpinus*	Li1478b	China	MW149435	MW151269	MW452610	Huang et al. 2021
*G. ammophilus*	AH:45814	Spain	KX869878	KX869892	-	Crous et al. 2016
*G. ammophilus*	AH:45842	Spain	KX869876	KX869890	-	Crous et al. 2016
*G. ammophilus*	AH:45843	Spain	KX869877	KX869891	-	Crous et al. 2016
*G. australiensis*	REH9312	Queensland	-	-	MF818180	Davoodian et al. 2018
*G. australiensis*	REH9559	Queensland	-	-	MF818182	Davoodian et al. 2018
*G. australiensis*	REH9492	Queensland	-	-	MF818181	Davoodian et al. 2018
*G. australiensis*	REH9501	Queensland	-	-	MF818183	Davoodian et al. 2018
*G. austrobrasiliensis*	ICN 184400	Brazil	MF437000	MF437015	-	Magnago et al. 2018
*G. austrobrasiliensis*	ICN 184402	Brazil	MF437001	OM068915	-	Magnago et al. 2018
*G. austrobrasiliensis*	ICN 184399	Brazil	MF436999	MF437014	-	Magnago et al. 2018
*G. austrocyanescens*	REH9700	Queensland	-	-	MF818176	Davoodian et al. 2018
*G. brunneofloccosus*	GDGM74550	China	ON502904	ON502927	ON100612	This study
*G. brunneofloccosus*	GDGM77131	China	ON502907	ON502930	ON100615	This study
*G. brunneofloccosus*	GDGM77125	China	ON502906	ON502929	ON100614	This study
*G. brunneofloccosus*	GDGM74638	China	ON502905	ON502928	ON100613	This study
*G. brunneofloccosus*	GDGM78301	China	ON502908	ON502931	ON100616	This study
*G. brunneofloccosus*	Wu2644a	China	MW149436	MW151267	MW452611	Huang et al. 2021
*G. brunneofloccosus*	HKAS107735	China	MW149436	-	-	Huang et al. 2021
*G. brunneofloccosus*	OR482	-	-	-	MF818146	Davoodian et al. 2018
*G*. aff. *castaneus*	CM061	Algeria	KP826761	-	-	Unpublished
*G*. aff. *castaneus*	E843c	-	-	EU718170	-	Wilson et al. 2012
*G*. cf. *castaneus*	FHMU3368	China	MW38086	MW352984	-	Xie et al. 2022
*G*. cf. *castaneus*	HKAS76672	China	-	KF112478	-	Unpublished
*G*. cf. *castaneus*	iNaturalist 31,940,211	USA	MN498109	-	-	Unpublished
*G. castaneus*	Arora 01 512	-	-	FJ710209	-	Unpublished
*G. castaneus*	FLAS F 61255	USA	MH211836	-	-	Unpublished
*G. castaneus*	SD Russell MycoMap 6269	USA	MK532856	-	-	Unpublished
*G. castaneus*	JMP0028	USA	EU819468	-	-	Unpublished
*G. castaneus*	F:PRL5664MAN	USA	-	GQ166887	-	Unpublished
*G. castaneus*	F:PRL5872MAN	USA	-	GQ166884	-	Unpublished
*G. castaneus*	F:PRL5948MAN	USA	-	GQ166885	-	Unpublished
*G. castaneus*	FLAS F 61844s	USA	MH212108	-	-	Unpublished
*G. castaneus*	FLAS F 61497	USA	MH211929	-	-	Unpublished
*G. castaneus*	ND31	USA	-	-	MF818163	Davoodian et al. 2018
*G. castaneus*	REH7761	Costa Rica	-	-	MF818162	Davoodian et al. 2018
*G. castaneus*	CS1	USA	-	-	MF818169	Davoodian et al. 2018
*G. castaneus*	MG531	Italy	-	-	MF818167	Davoodian et al. 2018
*G. castaneus*	VDKO979	Belgium	-	-	MF818168	Davoodian et al. 2018
*G. castaneus*	MG591	Italy	-	-	MF818189	Davoodian et al. 2018
*G. castaneus*	SW73	Pakistan	-	-	MF818184	Davoodian et al. 2018
*G. castaneus*	SW33	Pakistan	-	-	MF818164	Davoodian et al. 2018
*G. castaneus*	JFA13725	USA			MF818193	Davoodian et al. 2018
*G. castaneus*	NY1393558	USA	-	-	MF818187	Davoodian et al. 2018
*G. castaneus*	ND59WS	USA	-	-	MF818161	Davoodian et al. 2018
*G. castaneus*	ND58WS	USA	-	-	MF818160	Davoodian et al. 2018
*G. castaneus*	JPN12 770	Japan	-	-	MF818190	Davoodian et al. 2018
*G. castaneus*	TBG12 712	Japan	-	-	MF818188	Davoodian et al. 2018
*G. castaneus*	NY1782655	Italy	-	-	MF818186	Davoodian et al. 2018
*G*. aff. *cyanescens*	OKM23719	-	EU718104	EU718140	-	Unpublished
*G*. aff. *cyanescens*	REH8819	-	-	EU718172	-	Wilson et al. 2012
*G*. aff. *cyanescens*	E486	-	-	EU718173	-	Wilson et al. 2012
*G*. aff. *cyanescens*	E5685	-	-	EU718174	-	Wilson et al. 2012
*G. cyanescens*	0733	Japan	-	-	MF818191	Davoodian et al. 2018
*G. cyanescens*	AH46009	Spain	KY576810	KY576811	-	Unpublished
*G. cyanescens*	MCVE28580	Italy	KT363684	KT363685	-	Vizzini et al. 2015
*G. cyanescens*	17,184	Italy	JF908785	-	-	Osmundson et al. 2013
*G. cyanescens*	2837	-	KM248948	-	-	Unpublished
*G. cyanescens*	FLAS F 60581	USA	MH016792	-	-	Unpublished
*G. cyanescens*	FLAS F 61545	USA	MH211963	-	-	Unpublished
*G. cyanescens*	FLAS F 61592	USA	MH211984	-	-	Unpublished
*G. cyanescens*	FLAS F 61205	USA	MH211810	-	-	Unpublished
*G. cyanescens*	MB05-04	-	EU718102	-	-	Unpublished
*G. cyanescens*	NY1782681	Italy	-	-	MF818185	Davoodian et al. 2018
*G. cyanescens*	CNV67	USA	MT345244	-	-	Unpublished
*G. cyanescens*	0745	Japan	-	-	MF818192	Davoodian et al. 2018
*G. cyanescens*	ND11	USA	-	-	MF818173	Davoodian et al. 2018
*G. cyanescens*	REH9970	USA	-	-	MF818174	Davoodian et al. 2018
*G. cyanescens*	REH8758	-	-	EU718171	-	Wilson et al. 2012
*G. cyanescens*	MG639a	Italy	-	-	MF818172	Davoodian et al. 2018
*G. cyanescens var. cyanescens*	NAMA190	USA	EU819495	-	-	Palmer et al. 2008
*G. flavocyanescens*	WXL1182	China	MW440550	MW442950	MW452613	Huang et al. 2021
*G. flavocyanescens*	WXL1187	China	MW440551	MW442951		Huang et al. 2021
*G. flavocyanescens*	GDGM86062	China	-	ON505949	ON087646	This study
*G. furvescens*	REH9673	Queensland	-	-	MF818175	Davoodian et al. 2018
*G. lacteus*	MCVE:28582	Italy	KT363682	KT363683	-	Vizzini et al. 2015
*G. atrocyanescens*	GDGM75894	China	ON502909	ON502932	ON087647	This study
*G. atrocyanescens*	GDGM85841	China	-	ON502934	ON087648	This study
*G. atrocyanescens*	GDGM83673	China	-	ON502933	-	This study
*G. atrocyanescens*	GDGM76540	China	-	ON502910	-	This study
*G. longicystidiatus*	GDGM25857	China		-	ON087649	This study
*G. longicystidiatus*	OR74	Thailand	-	-	MF818152	Davoodian et al. 2018
*G. longicystidiatus*	OR394	Thailand	-	-	MF818153	Davoodian et al. 2018
*G. longicystidiatus*	GDGM46175	China	ON502912	ON502936	ON087650	This study
*G. longicystidiatus*	GDGM42941	China	ON502911	ON502935	ON087652	This study
*G. longicystidiatus*	GDGM52128	China	ON502913	ON502937	ON087651	This study
*G. longicystidiatus*	EN99-67	Japan	-	-	MF818151	Davoodian et al. 2018
*G. longicystidiatus*	OR235	China	-	-	MF818202	Davoodian et al. 2018
*G. longicystidiatus*	OR238	China	-	-	MF818155	Davoodian et al. 2018
*G. longicystidiatus*	FHMU1997	China	MW380860	MW352983	-	Xie et al. 2022
*G. longicystidiatus*	FHMU2234	China	-	MW352966	-	Xie et al. 2022
*G. longicystidiatus*	FHMU3367	China	-	MW352970	-	Xie et al. 2022
*G. longicystidiatus*	FHMU1935	China	MW380859	MW352982	-	Xie et al. 2022
*G. longicystidiatus*	FHMU900	China	MW380852	MW352975	-	Xie et al. 2022
*G. longicystidiatus*	FHMU954	China	MW380857	MW352980	-	Xie et al. 2022
*G. longicystidiatus*	FHMU1582	China	MW380845	MW352965	-	Xie et al. 2022
*G. longicystidiatus*	FHMU3366	China	MW380849	MW352971	-	Xie et al. 2022
*G. longicystidiatus*	REH8799	Thailand	EU718142	EU718106	MF818147	Davoodian et al. 2018
*G. mcnabbii*	E8155	USA	-	EF561627	MF818195	Davoodian et al. 2018
*G. mcnabbii*	REH9808	Queensland	-	-	MF818197	Davoodian et al. 2018
*G. mcnabbii*	REH8955	Queensland	-	-	MF818198	Davoodian et al. 2018
*G. memnonius*	GDGM44779	China	ON502914	ON502938	ON087653	This study
*G. memnonius*	GDGM78781	China	ON502915	ON502939	ON087654	This study
*G. memnonius*	FHMU3369	China	MW380858	MW352981	-	Xie et al. 2022
*G. memnonius*	FHMU929	China	MW380856	MW352979	-	Xie et al. 2022
*G. naranjus*	REH9020	Queensland	-	-	MF818158	Davoodian et al. 2018
*G. naranjus*	REH9411	Queensland	-	-	MF818157	Davoodian et al. 2018
*G. occidentalis*	E8164	USA	-	-	MF818194	Davoodian et al. 2018
*G. occidentalis*	REH8821	Australia	EU718103	EU718139	MF818177	Davoodian et al. 2018
*G. pallidus*	GDGM46275	China	ON502918	ON502942	ON087657	This study
*G. pallidus*	GDGM46401	China	ON502920	-	ON087659	This study
*G. pallidus*	GDGM46405	China	ON502921	ON502944	ON087660	This study
*G. pallidus*	GDGM46509	China	-	ON505947	ON087663	This study
*G. pallidus*	GDGM46387	China	ON502919	ON502943	ON087658	This study
*G. pallidus*	GDGM46419	China	ON502922	ON502945	ON087661	This study
*G. pallidus*	GDGM46433	China	ON502923	ON502946	ON087662	This study
*G. paralongicystidiatus*	NY48429	Colombia	-	-	MF818148	Davoodian et al. 2018
*G. paralongicystidiatus*	REH8274	Costa Rica	-	-	MF818150	Davoodian et al. 2018
*G. paralongicystidiatus*	REH7725	Costa Rica	-	-	MF818149	Davoodian et al. 2018
*G. paramjitii*	FHMU2243	China	MW380847	MW352968	-	Xie et al. 2022
*G. paramjitii*	FHMU2240	China	MW380846	MW352967	-	Xie et al. 2022
*G. paramjitii*	GDGM52188	China	ON502917	ON502941	-	This study
*G. paramjitii*	CAL KD 162–002	India	MF120284	MF120285	-	Das et al. 2017
*G. paramjitii*	HKAS63505	China	-	KF112476	-	Wu et al. 2014
*G. phaeocyanescens*	ARB1309	USA	-	-	MF818144	Davoodian et al. 2018
*G. porphyreus*	FHMU917	China	MW380854	MW352977	-	Xie et al. 2022
*G. porphyreus*	FHMU926	China	MW380855	MW352978	-	Xie et al. 2022
*G. porphyreus*	FHMU888	China	MW380850	MW352973	-	Xie et al. 2022
*G. porphyreus*	FHMU2273	China	MW380848	MW352969	-	Xie et al. 2022
*G. porphyreus*	FHMU905	China	MW380853	MW352976	-	Xie et al. 2022
*G. pseudocyanescens*	AH55729	Spain	KY576808	KY576806	-	Unpublished
*G. pseudocyanescens*	AH45840	Spain	KY576809	KY576807	-	Unpublished
*G. pseudocyanescens*	ECC17070501	Spain	MW376657	-	-	Unpublished
*G. pseudolongicystidiatus*	GDGM42787	China	ON502916	ON502940	ON087655	This study
*G. pseudolongicystidiatus*	GDGM42986	China	-	ON505946	ON087656	This study
*G. pseudolacteus*	AH45850	Spain	KX869871	KX869885	-	Crous et al. 2016
*G. pseudolacteus*	AH45849	Spain	KX869868	KX869882	-	Crous et al. 2016
*G. pseudolacteus*	AH39364	Spain	KX869866	KX869880	-	Crous et al. 2016
*G. pseudolacteus*	AH44522	Spain	KX869873	KX869887	-	Crous et al. 2016
*G. pseudolacteus*	AH45812	Spain	KX869870	KX869884	-	Crous et al. 2016
*G. pseudolacteus*	AH45848	Spain	KX869867	KX869881	-	Crous et al. 2016
*G. pseudolacteus*	AH37878	Spain	KX869872	KX869886	-	Crous et al. 2016
*G. pseudolacteus*	AH45811	Spain	KX869869	KX869883	-	Crous et al. 2016
*G. pseudolacteus*	HaI BP16	Spain	MT594507	-	-	Leonardi et al. 2020
*G. purpurinus*	Chpn776	USA	KX389110	-	-	Unpublished
*G. purpurinus*	PRL3737	-	EU718105	EU718141	-	Wilson et al. 2012
*G. robinsonii*	ND13	USA	-	-	MF818178	Davoodian et al. 2018
*G. smithii*	REH4511	USA	-	-	MF818159	Davoodian et al. 2018
*G. smithii*	ND57	USA	-	-	MF818165	Davoodian et al. 2018
*G. smithii*	MICH232867	USA	-	-	MF818166	Davoodian et al. 2018
*G*. sp.	OR182	Thailand	-	-	MF818156	Davoodian et al. 2018
*G*. sp.	BOS472	BLZ	-	-	MF818196	Davoodian et al. 2018
*G*. sp.	TH9913	CMRN	-	-	MF818170	Davoodian et al. 2018
*G*. sp.	Thoen7634	SEN	-	-	MF818171	Davoodian et al. 2018
*G*. sp.	Arora00 429	-	EU718107	EU718143	-	Wilson et al. 2012
*G*. sp.	Arora14800	USA	MW343686	-	-	Unpublished
*G*. sp.	E4879c	-	-	FJ710208	-	Wilson et al. 2012
*G*. sp.	JLF8835	USA	MW343688	-	-	Unpublished
*G*. sp.	JLF8747	USA	MW343687	MW341339	-	Unpublished
*G. subalbellus*	OKM25477	USA	EU718108	EU718144	-	Wilson et al. 2012
*G*. aff. *subalbellus*	HONDURAS19	USA	MT571529	-	-	Haelewaters et al. 2021
*G. subcaerulescens*	GDGM60494-1	China	ON502924	ON502947	ON087665	This study
*G. subcaerulescens*	GDGM60494-2	China	-	-	ON087664	This study
*G. subglobosus*	FHMU3364	China	-	MW352985	-	Xie et al. 2022
*G. subglobosus*	FHMU859	China	MW380851	MW352974	-	Xie et al. 2022
*G. umbrinisquamosus*	BUF-Both3525	USA	-	-	MF818145	Davoodian et al. 2018
*Phlebopus spongiosus*	CMUB39824	Thailand	KX575660	KX575655	-	Thongkantha et al. 2021
*Ph. spongiosus*	BC0166	Thailand	-	MT757956	MT755374	Unpublished

Phylogenetic analyses were performed in the software of PhyloSuite (Zhang et al. 2020). Maximum likelihood phylogenies were inferred using IQ-TREE (Nguyen et al. 2015) under the TPM2u+R3 + F model for 5000 ultrafast bootstraps, as well as the Shimodaira-Hasegawa-like approximate likelihood-ratio test. Bayesian Inference (BI) phylogenies were inferred using MrBayes 3.2.6 (Ronquist et al. 2012), the best models for the combined datasets ITS-nrLSU were searched via PartitionFinder 2 (Lanfear et al. 2017), and for *atp6* region was searched via ModelFinder (Kalyaanamoorthy et al. 2017). BI analysis using 4 chains were conducted by setting generations to 20 million and stoprul command with the value of stopval set to 0.01, trees were sampled every 1000 generations, the first 25% generations were discarded as burn-ins and posterior probabilities (PP) were then calculated from the posterior distribution of the retained Bayesian trees. The phylogenetic trees were visualised using FigTree v1.4.23.

## Results

### Molecular phylogeny

**Figure 1.**
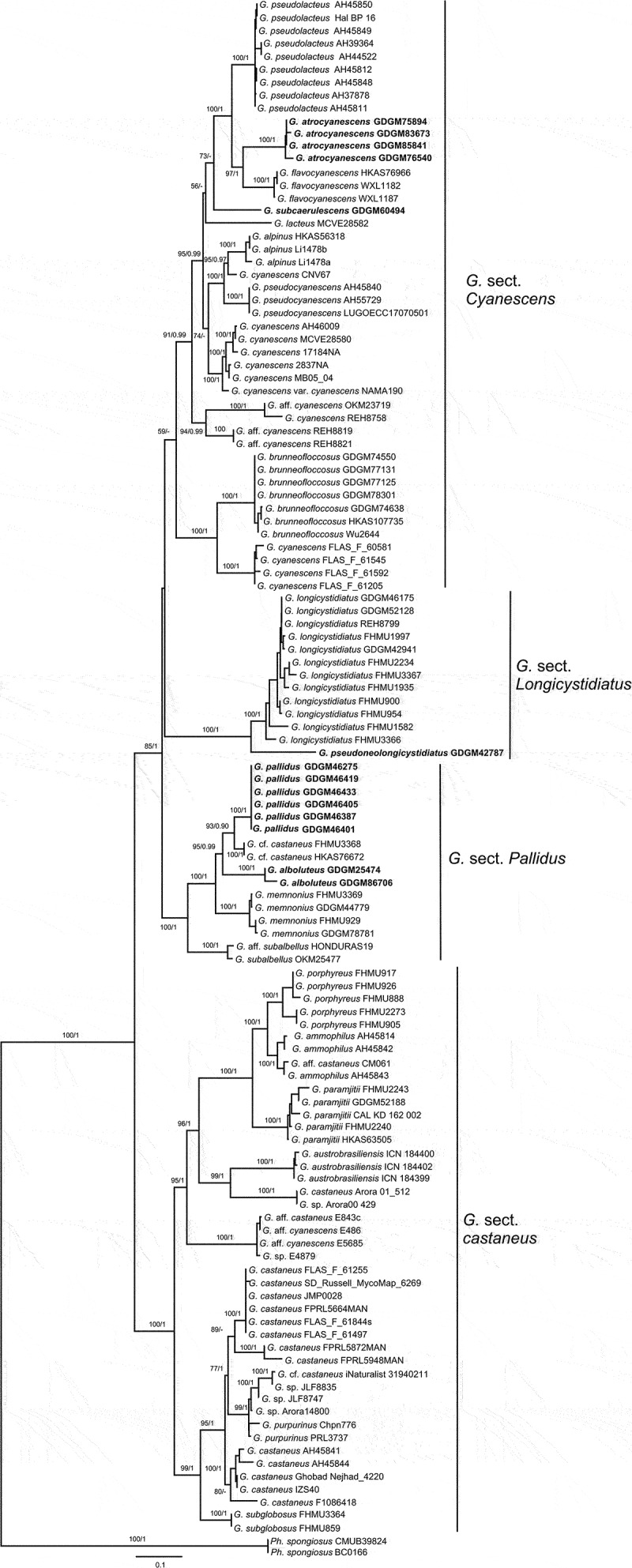
Maximum likelihood phylogenetic tree of *Gyroporus* inferred from the ITS-nrLSU dataset. Bootstrap frequencies (> 50%) and Bayesian posterior probabilities (BPP > 0.90) are shown above or below supported branches. New species are indicated in bold.

**Figure 2.**
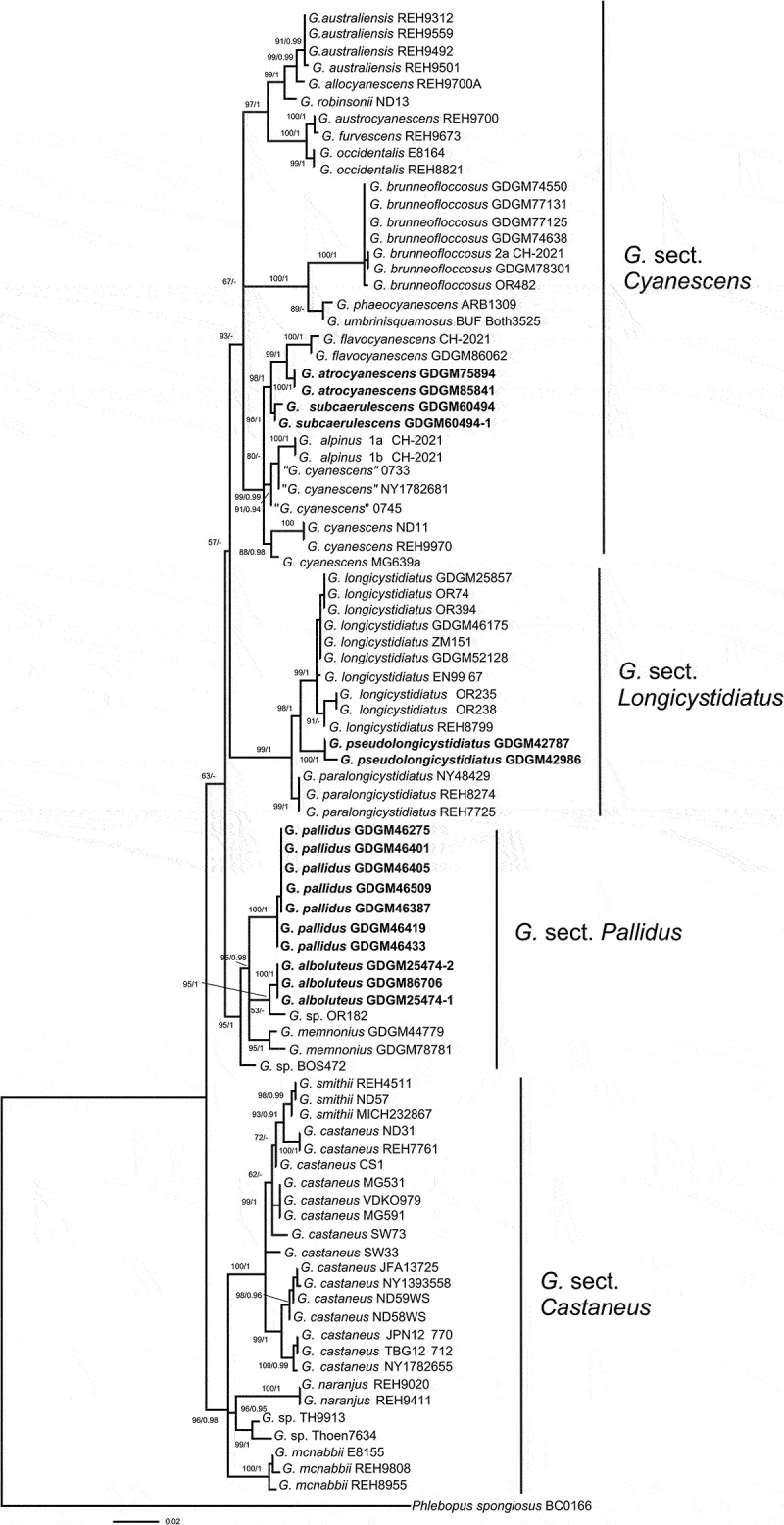
Maximum likelihood phylogenetic tree of *Gyroporus* inferred from the *atp6* dataset. Bootstrap frequencies (> 50%) and Bayesian posterior probabilities (BPP > 0.90) are shown above or below supported branches. New species are indicated in bold.

In the concatenated (nrLSU + ITS) dataset, 188 sequences (95 for nrLSU and 88 for ITS) from 121 fungal collections were included, including 39 sequences newly generated in this study. The alignment length was 1816 characters including gaps (888 characters for ITS, and 928 characters for nrLSU), TVM + I + G and TIM + I + G were selected for ITS and nrLSU respectively for the BI analysis. In the *atp*6 dataset, 89 sequences were included, including 23 sequences newly generated in this study. The alignment length was 616 characters. GTR + F + I + G4 was selected as the best models for Bayesian inference. *Phlebopus spongiosus* Pham & Har. Takah. was selected as outgroup based on recently studies (Davoodian et al. 2018; Xie et al. 2022). The tree topologies obtained by ML and Bayesian analyses were similar; thus, only the ML topology was shown in Figures 1 and 2. Phylogenetic analyses showed that *Gyroporus* was supported as a monophyletic group, and five new lineages were discovered in present study (Figures 1 and 2). Species of *Gyroporus* formed four main branches, and four new sections were firstly proposed herein, i.e. “*G*. sect. *Castaneus*”, “*G*. sect. *Cyanescens”*, “*G*. sect. *Longicystidiatus”* and “*G*. sect. *Pallidus”. Gyroporus* sect. *Castaneus* was well-supported as a monophyletic group, and located at the base of the phylogenetic trees. *Gyroporus* sect. *Cyanescens* formed an independent branch, but with moderate support in the *atp*6 tree, and weak support in the ITS-nrLSU tree. Two new species *G. atrocyanescens* and *G. subcaerulescens* nested into the sect. *Cyanescens. Gyroporus atrocyanescens* was well-supported as an independent clade in the phylogenetic trees (Figures 1 and 2), and formed sister relationship with *G. flavocyanescens. Gyroporus subcaerulescens* formed an independent clade in Figure 1, while clustered together with *G. atrocyanescens* and *G. flavocyanescens* in Figure 2. The sect. *Longicystidiatus* was well-supported in the phylogenetic trees, and three species were included, containing a new species discovered in present study. The sect. *Pallidus* was well-supported as an independent branch in the trees (Figures 1 and 2), and different from the sect. *Castaneus*. Two new species *G. alboluteus* and *G. pallidus* nested into the branch, and close to “*G*. cf. *castaneus”* and *G. memnonius*.

### Taxonomy

*Gyroporus* section *Castaneus* Ming Zhang & T.H. Li sect. nov.

Fungal Name: FN570996

Type species: *Gyroporus castaneus* (Bull.) Quél., Enchir. fung. (Paris): 161 (1886)

Etymology: “*castaneus”* refers to the species in this section similar to *G. castaneus.*

Basidiomata small to medium-sized. Pileus hemispheric, convex to applanate, dry, subtometosus, yellow-brown, brownish orange, brown, dark brown to red brown, usually with red or purple tinge; context white, unchanging when injured. Hymenophore poroid, white, unchanging when bruised. Stipe central, surface dry, glabrous or subtomentosus, unchanging when handled; basal mycelium white; annulus absent. Basidiospores oval to ellipsoid, thin-walled, smooth. Basidia clavate, thin-walled, 4-spored, hyaline in 5% KOH. Hymenophoral trama composed of thin- to thick-walled hyphae. Cheilocystidia subfusiform or fusiform, thin-walled. Pleurocystidia absent or present. Pileipellis a trichodermium, composed of thin to thick-walled hyphae. Clamp connections frequently present in all tissues.

Notes: *Gyroporus* sect. *Castaneus* as a monophyletic branch is strongly supported in our phylogenetic analyses (Figures 1 and 2, BS/BPP = 96%/0.98; BS/BPP = 100%/1). Species in this section are mainly characterised by their brown to yellowish brown pileus, usually with red or purple tinge, white context unchanging when bruised, oval to elliptical basidiospores, and a trichoderm pileipellis composed of clavate to subcylindrical hyphae. Seven species, *G. castaneus, G. mcnabbii* Davoodian, Bougher & Halling, *G. naranjus* Davoodian, Bougher, Fechner & Halling, *G. paramjitii, G. porphyreus, G. purpurinus* Singer ex Davoodian & Halling, and *G. subglobosus* have been proved to belong to this section based on the morphological features and phylogenetic analyses.

*Gyroporus* section *Cyanescens* Ming Zhang & T.H. Li sect. nov.

Fungal Name: FN570997

Type species: *Gyroporus cyanescens* (Bull.) Quél., Enchir. fung. (Paris): 161 (1886)

Etymology: “*cyanescens”* refers to the species in this section usually with cyanescent oxidation reactions similar to that of *G. cyanescen*s.

Basidiomata medium to large-sized. Pileus hemispherical to convex, dry, greyish yellow, greyish orange, brown or red-brown, covered with floccose-scaly to coarsely tomentose squamules; context white, becoming bluish, greenish blue or dark blue or deep blue when bruised. Hymenophore poroid, white, yellowish, to greenish-yellow, becoming bluish, greenish blue or dark blue when bruised. Stipe central, dry, covered with tomentose to fibrillose squamules, unchanging or changing to blue when handled; basal mycelium white; annulus indistinct to as a weak annular zone. Basidiospores ellipsoid to broadly ellipsoid, smooth, yellowish in 5% KOH. Basidia clavate, 4-spored, hyaline in 5% KOH. Cheilocystidia clavate to subfusiform, thin-walled, yellowish to hyaline in 5% KOH. Pleurocystidia absent or present. Pileipellis a trichodermium, composed of elongated or somewhat clumped, parallel to slightly interwoven, thin to thick-walled hyphae, colourless or yellowish in 5% KOH. Stipitipellis composed of thin- to thick-walled hyphae, colourless to yellowish. Clamp connections frequently present in all tissues.

Notes: *Gyroporus* sect. *cyanescens* formed an independent branch in the phylogenetic trees (Figures 1 and 2), but with moderate support at *atp6* tree and weak supported at ITS-nrLSU tree. Morphologically, species in this section all can produce cyanescent oxidation reactions, and pileus surface always covered with elongated and somewhat clumped tomentum. Fourteen species were proved to belong to this section, including two new species *G. atrocyanescens* and *G. subcaerulescens* discovered in present study.

**Figure 3.**
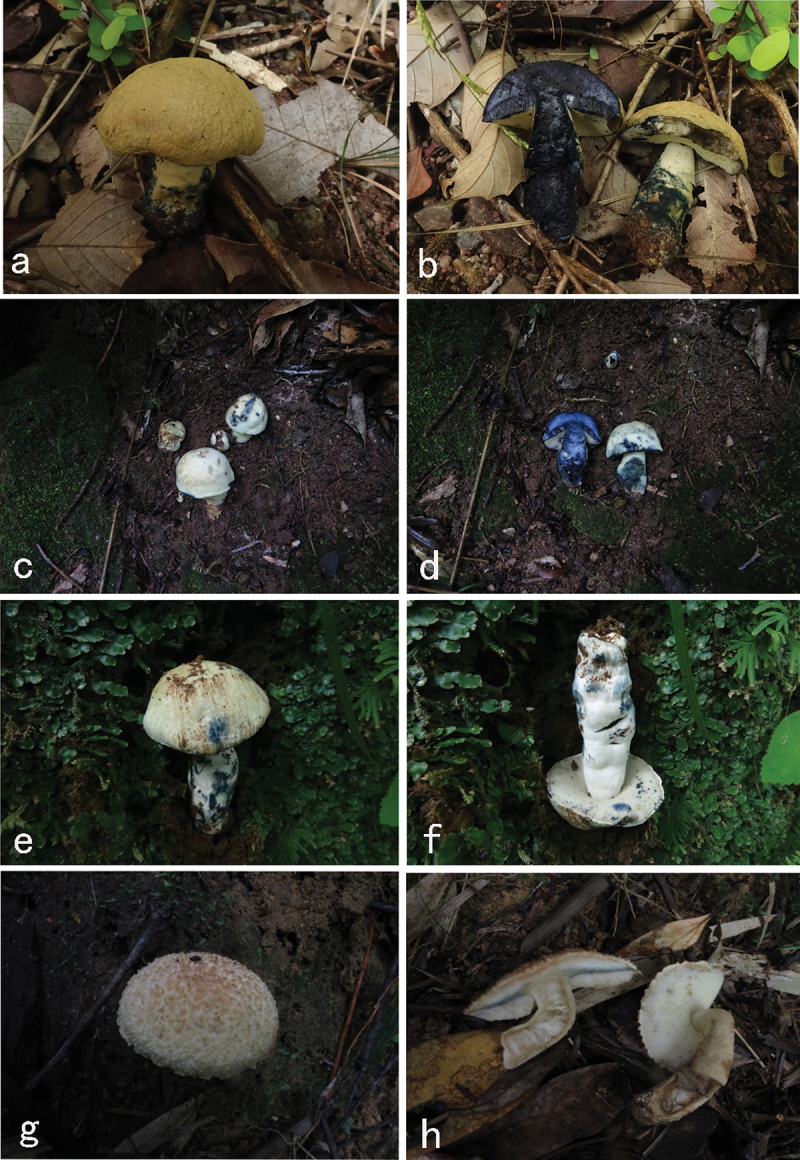
Basidiomata of *Gyroporus* species. a–f. *Gyroporus atrocyanescens* (a–b from GDGM75894; c–d from GDGM85841; e–f from GDGM83673). g–h *Gyroporus subcaerulescens* (GDGM70494).

**Figure 4.**
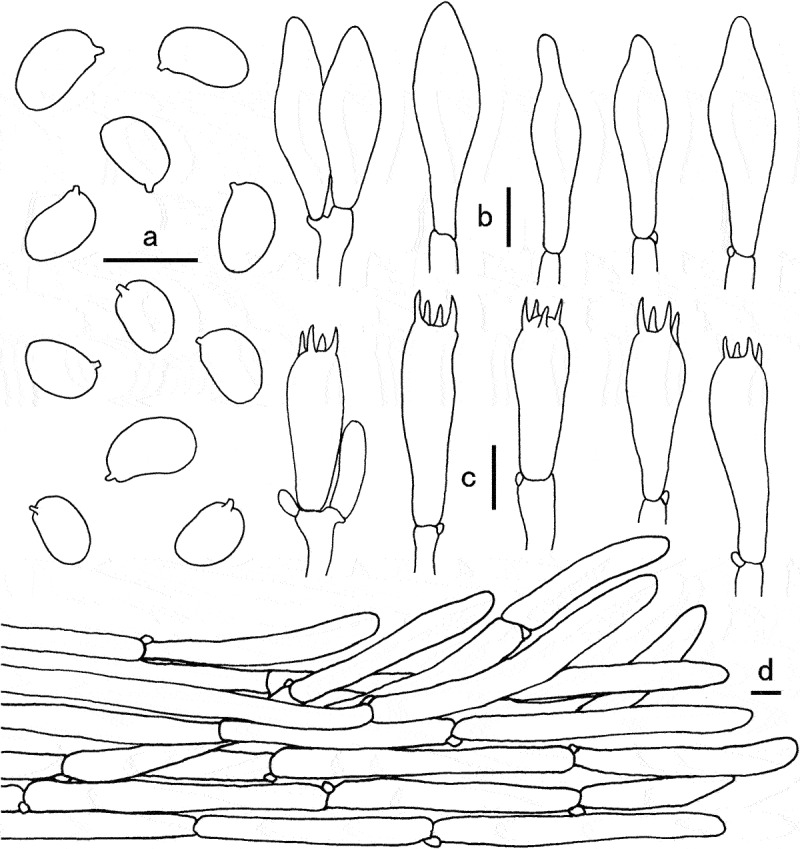
Microscopic features of *Gyroporus atrocyanescens*. a. Basidiospores; b. Cheilocystidia; c. Basidia; d. Pileipellis. Bras = 10 μm.

*Gyroporus atrocyanescens* Ming Zhang & T.H. Li sp. nov. Figures 3a–f, 4

Fungal Name: FN570980

Etymology: “*atro-*” means black, “*cyanescens*” means becoming blue, “*atrocyanescens*” refers to the basidiomata instantly changing to blackish blue when bruised.

Diagnosis: This species is characterised by its white to greyish yellow pileus densely covered with greyish yellow floccose squamules, white to yellowish white hymenophore, broadly elliptical basidiospores (7.5–10 × 4.8–6 μm), and the whole basidiomata immediately staining dull blue, deep blue to dark blue when bruised.

Holotype: CHINA. Guangdong Province, Shaoguan City, Renhua County, Danxiashan National Natural Reserve, alt. 300 m, 27 September 2018, Xiang-Rong Zhong (GDGM75894).

Basidiomata medium sized. Pileus 4–6 cm broad, sub-hemispherical to convex when young, broadly convex to nearly applanate at maturity, dry, white to yellowish white when young, dull yellow, olive yellow to greyish yellow (3B3–4B3, 3B4–4B4, 3C6) when mature; densely covered with greyish yellow (4B3) appressed scales to floccose squamules; margin incurved and slightly extended, usually cracked at maturity. Context white (1A1), 8–15 mm thick at pileus centre, immediately and intensely staining deep blue (19D8–21D8), blackish blue to dark blue (19F8–21F8) when bruised. Hymenophore adnate to slightly depressed around stipe when mature, 3–5 mm long, white (1A1) when young, yellowish white (3A2) when mature, staining deep blue (19D8–21D8), blackish blue to dark blue (19F8–21F8) when bruised; pores angular to roundish, 3–4 per mm, staining deep blue to dark blue when bruised. Stipe 5–7 × 1.5–2.5 cm, central, sub-cylindrical to clavate, white (2A1) when young, yellowish-white (2A2–4A2) when mature; surface rough, staining dull blue to greyish blue when bruised (22D5–23D5); context white to yellowish white, spongy when young and then hollow in age, staining deep blue to dark blue when bruised. Odour none. Taste mild.

Basidiospores (7.5)8–10(10.5) × (4.5)4.8–6.5(7) µm, [Q = (1.5)1.54–1.7(1.8), Q_m_ = 1.65 ± 0.09], smooth, ellipsoid to somewhat broadly ellipsoid, yellowish in 5% KOH. Basidia 24–35 × 8–10 µm, clavate, 4-spored, hyaline in 5% KOH. Cheilocystidia 28–40 × 8–12 µm, clavate to subfusiform, thin-walled, vivid yellow in 5% KOH at first, then hyaline. Pleurocystidia not observed. Tube trama composed of 4–10 µm wide parallel hyphae, hyaline to yellowish in 5% KOH. Pileipellis a cutis, composed of 8–16 µm wide, repent to suberect, parallel to slightly interwoven hyphae, thin-walled, hyaline to yellowish in 5% KOH; terminal cells 60–130 × 8–14 µm, clavate to subcylindrical, obtuse at apex. Pileal trama made up of hyphae 6–18 μm broad, hyaline in 5% KOH. Stipitipellis composed of thin-walled hyphae, 5–15 μm wide, light yellow in 5% KOH. Stipe trama composed of cylindrical, light yellow in 5% KOH, thin-walled, interwoven hyphae 5–16 μm wide. Clamp connections frequently present in all tissues.

Additional specimens examined: CHINA. Guangdong Province, Shaoguan City, Renhua County, Danxiashan National Natural Reserve, alt. 300 m, 26 June 2021, Ming Zhang (GDGM85841); same location, alt. 350 m, 24 September 2021, Guo-Rui Zhong (GDGM83673); same location, alt. 330 m, 15 May 2019, Juan-Yan Xu (GDGM76540).

Habitat and distribution: Solitary or scattered on soil in subtropical broad-leaf forest dominated by Fagaceae trees. Currently known only from southern China.

Notes: Phylogenetic analyses showed that *G. atrocyanescens* was nested into the sect. *Cyanescens*, and closely related to *G. flavocyanescens*. However, the latter species, recently reported from southwestern China, differs in its larger basidiomata, dull yellow to greyish-orange pileus, nearly glabrous or somewhat fibrillose to finely tomentose pileal surface, broader basidiospores (8–10 × 5.5–6.5 µm) and hyaline cheilocystidia in 5% KOH (Huang et al. 2021).

In morphology, *G. occidentalis* Davoodian, Bougher & Halling resembles *G. atrocyanescens* in the rapidly bluing oxidation reaction. However, *G. occidentalis*, reported from Western Australia, differs in its larger basidiomata, yellow-white to yellow buff to dirty yellow pileus, and smaller and narrower basidiospores 7.7–8.4(9.1) × 3.5–4.2 µm (Davoodian et al. 2018). The bluing species *G. alpinus, G. brunneofloccosus* and *G. cyanescens* are also similar to *G. atrocyanescens* in the discolouration. However, *G. alpinus* recently reported from southwestern China, differs in its ivory yellow to brownish-yellow pileus covered with concolourous appressed scaly to floccose squamules, broader basidiospores (5.5–8.5 µm broad), and distribution in alpine forests dominated by *Abies, Picea* and *Quercus* (Huang et al. 2021); *G. brunneofloccosus*, reported from subtropical regions of southern China, differs in its dark brown to light red brown pileus covered with concolourous floccose-scaly to coarsely tomentose squamules, yellowish to greenish-yellow hymenophore staining cerulean blue to greenish-blue when bruised, brownish to light red-brown stipe, and clavate to subfusiform cheilocystidia hyaline in 5% KOH (Li et al. 2003; Huang et al. 2021); while *G. cyanescens*, originally described from Europe, differs in its larger basidiomata, pale straw, buff to ivory pileus covered with obviously fibrillose tomentum, more robust stipe with a pseudo-annular zone and horizontal fissures at stipe apex, and distribution in forests dominated by *Pinus sylvestris* or *Fagus sylvatia* (Watling 1970; Vizzini et al. 2015).

**Figure 5.**
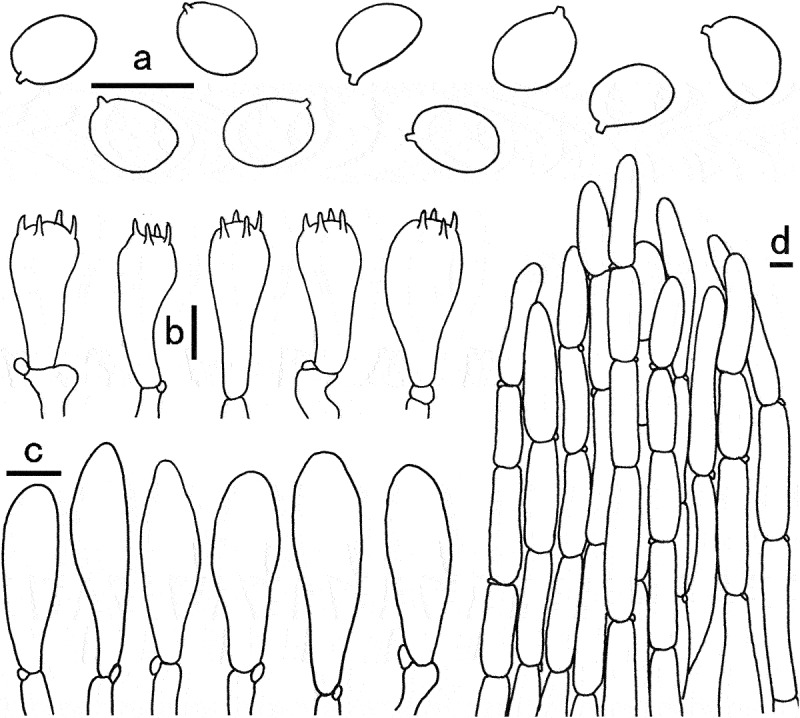
Microscopic features of *Gyroporus subcaerulescens*. a. Basidiospores; b. Basidia; c. Cheilocystidia; d. Pileipellis. Bras = 10 μm.

*Gyroporus subcaerulescens* Ming Zhang & T.H. Li sp. nov. Figures 3g–h, 5

Fungal Name: FN570983

Etymology: “*subcaerulescens*” means “becoming pale blue or blueish”, refers to the context slightly changing to bluish when exposed.

Diagnosis: This species is characterised by its white to orange white pileus covered with orange white to reddish white coarsely tomentose squamules, white hymenophore and pileus context slowly changing to pastel blue when bruised, elliptical basidiospores (6.5) 8–10 × 5.5–6.5 (7.0) µm.

Holotype: CHINA. Hunan Province, Chenzhou City, Yizhang County, Mangshan National Natural Reserve, alt. 1000 m, 30 July 2017, Hao Huang (GDGM70494).

Basidiomta small to medium sized. Pileus 3–5.8 cm broad, sub-hemispherical to convex when young, broadly convex to nearly applanate at mature, surface dry, white to orange white (5A1, 5A2–6A2), densely covered with orange white to reddish white (5A2–8A2) floccose scales to coarsely tomentose squamules, paler towards margin, margin incurved and slightly extended, usually cracked at age; context white (1A1), slowly staining pale blue to pastel blue (22A4–23A4) when bruised. Hymenophore adnate to slightly depressed around stipe when mature, 5–7 mm long, white (1A1) when young, yellowish white (1A2–2A2) when mature, unchanging when bruised; pores angular to roundish, 2–3 per mm, staining bluish white when bruised. Stipe 4–6 × 1–1.6 cm, sub-cylindrical to clavate, white (2A1) when young, yellowish-white (2A2) to concolourous with pileal surface when mature; surface roughened, unchanging when bruised; context white to cream or yellowish, spongy when young and then hollow in age, unchanging when bruised. Odour none. Taste mild.

Basidiospores (6.5) 8–10 × 5.5–6.5 (7.0) µm, Q = (1.28)1.35–1.64 (1.81), Q_m_ = 1.48 ± 0.13, smooth, elliptical, oval, to somewhat oblong, yellowish in 5% KOH. Basidia 23–32 × 9–14 µm, clavate, 4-spored, hyaline in 5% KOH. Cheilocystidia 31–45 × 9–15 µm, clavate to subfusiform, thin-walled, hyaline to yellowish in 5% KOH; Pleurocystidia not observed. Tube trama composed of interwoven hyphae, 5–13 µm wide, hyaline to yellowish in 5% KOH. Pileipellis a trichoderm, composed of erect, parallel to somewhat clumped hyphae, 8–22 µm wide, hyaline to yellowish in 5% KOH; terminal cells 55–130 × 8–22 µm, clavate to subcylindrical, with obtuse apex. Pileal trama made up of hyphae 6–20 μm diam, colourless in 5% KOH. Stipitipellis composed of thin-walled hyphae, 5–10 μm wide, light yellow in 5% KOH. Stipe trama composed of cylindrical, light yellow in 5% KOH, thin-walled, interwoven hyphae 5–10 μm wide. Clamp connections frequently present in all tissues.

Habitat and distribution: Solitary or scattered on soil in subtropical mixed forest mainly dominated by Fagaceae trees, with a few pine trees (*Cunninghamia* sp.). Currently known from Hunan Province, China.

Notes: Phylogenetic analyses shown that *G. subcaerulescens* was well nested into the sect. *Cyanescens*, and closely related to *G. alpinus, G. cyanescens, G. flavocyanescens* and *G. atrocyanescens*. However, they can be separated from each other by the genetic distance. Additionally, *G. alpinus*, recently reported from alpine forests of China, differs in its ivory yellow to brownish-yellow pileus densely covered with concolourous appressed floccose squamules, and broader basidiospores (6.5) 7–10 × 5.5–7.5 (8.5) µm (Huang et al. 2021); *G. cyanescens*, originally reported from Europe, differs in its larger basidiomata can up to 12 cm broad, pale straw pileus, larger but narrow basidiospores (9–11 × 4.5–6 µm) (Watling 1970; Vizzini et al. 2015; Huang et al. 2021); *G. flavocyanescens*, recently reported from tropical forests of China, differs in its larger basidiomata can up to 10 cm broad, nearly glabrous and flavous to greyish-orange pileus, white context staining strong dark blue when bruised, white to yellowish hymenophore staining cyanine blue to porcelain blue when bruised (Huang et al. 2021); *G. atrocyanescens*, newly described in this study, can be easily distinguished by its strongly cyanescent oxidation reactions.

In morphology, *G. subcaerulescens* resembles *G. robinsonii* with the slowly and faintly bluing oxidation reaction. However, *G. robinsonii* reported from Western Australia, differs in its yellow-white to dirty buff pileus, large and narrow basidiospores (8.4)8.8–10.5(12) × 4.7–5.6(6) µm (Davoodian et al. 2019). *Gyroporus brunneofloccosus*, reported from southern China, is also similar to *G. subcaerulescens* in sharing with the pileus covered with floccose scales to coarsely tomentose squamules, but differs in its larger basidiomata can up to 9 cm broad, darker brown to reddish brown pileus, white context staining cerulean blue to dark blue when bruised, and greenish-yellow hymenophore staining cerulean blue to greenish-blue when bruised (Li et al. 2003; Huang et al. 2021).

*Gyroporus* section *Longicystidiatus* Ming Zhang & T.H. Li sect. nov.

Fungal Name: FN570998

Type species: *Gyroporus longicystidiatus* Nagas. & Hongo, in Nagasawa, Rep. Tottori Mycol. Inst. 39: 18 (2001)

Etymology: “*longicystidiatus”* refers to the longer cheilo- or pleurocystidia.

Basidiomata medium-sized. Pileus hemispheric to convex, dry, subtomentose or glabrous, greyish orange, brownish orange, yellowish brown, dark brown; context white, unchanging when bruised. Hymenophore poroid, white, yellowish to greenish-yellow, unchanging when bruised. Stipe central, surface dry, glabrous to subtomentose unchanging when handled; basal mycelium white; annulus absent. Basidiospores elliptic, cylindrical to oblong, smooth, thin-walled, yellowish in 5% KOH. Basidia clavate, 4-spored, hyaline in 5% KOH. Cheilocystidia narrowly fusoid to cylindrical, can up to 100 μm long, thin-walled. Pleurocystidia present or absent. Pileipellis a trichodermium, composed of elongated, interwoven, thin to thick-walled hyphae. Clamp connections frequently present in all tissues.

Notes: *Gyroporus* sect. *Longicystidiatus* was well-supported as a monophyletic clade in our phylogenetic analyses (Figures 1 and 2; BS/BPP = 99%/0.98; BS/BPP = 100%/1). Species in this section mainly characterised by their brownish orange to brownish yellow pileus, white context unchanging when bruised, longer cystidia can up to 100 μm, and trichoderm pileipellis. Three species were included in this section, containing a new species described as follow.

**Figure 6.**
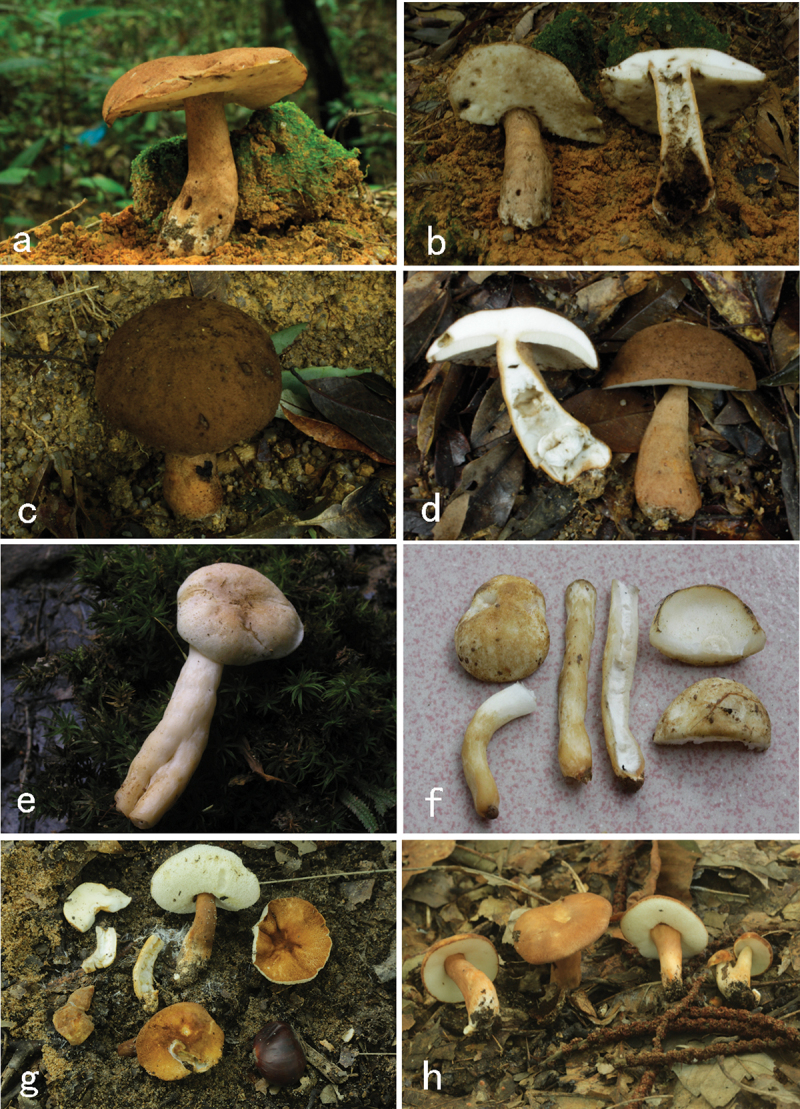
Basidiomata of *Gyroporus* species. a–d. *Gyroporus pseudolongicystidiatus* (a–b from GDGM42787; c–b from GDGM42986); e–f. *Gyroporus alboluteus* (e from GDGM86706; f from GDGM25474); g–h. *Gyroporus pallidus* (g from GDGM46387; h from GDGM46275).

**Figure 7.**
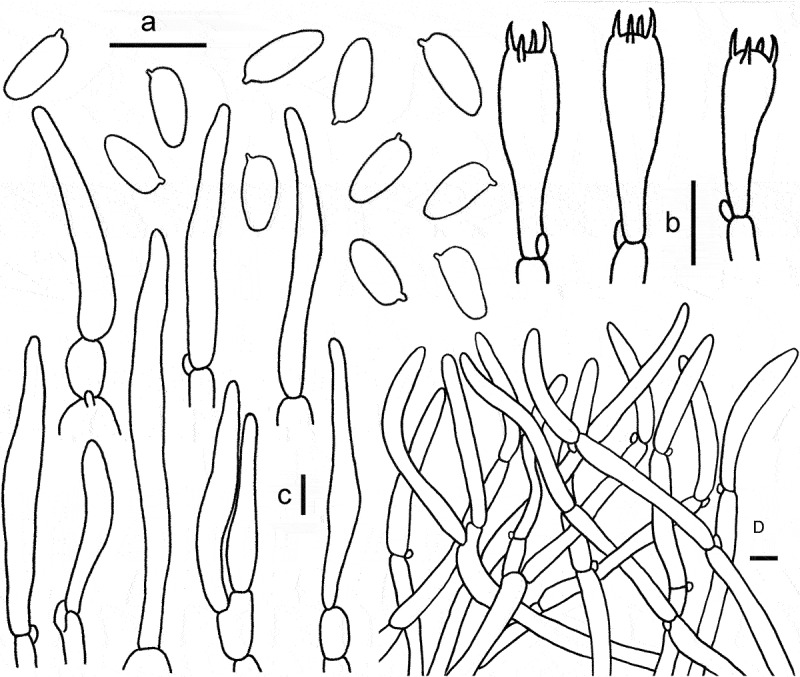
Microscopic features of *Gyroporus pseudolongicystidiatus*. a. Basidiospores; b. Basidia; c. Cheilocystidia. Bras = 10 μm.

*Gyroporus pseudolongicystidiatus* Ming Zhang, D.C. Xie & T.H. Li sp. nov. Figures 6a–d, 7

Fungal Name: FN570981

Etymology: “*pseudolongicystidiatus*” refers to the species similar to *G. longicystidiatus*.

Diagnosis: This species is characterised by its brownish orange to brownish yellow pileus subglabrous when mature, white hymenophore and pileus context unchanging when bruised, and elliptical to cylindrical basidiospores (6.5) 8–10 × 5.5–6.5 (7.0) µm.

Holotype: CHINA. Hainan, Ledong County, Jianfengling National Forest Park, at 18°44′N, 108°52′E, alt. 940 m, 3 July 2013, Ming Zhang (GDGM42787).

Basidiomata small to medium. Pileus 5–10 cm broad, hemispheric, convex to plane, fleshy, surface dry, fibrillose, velvet-subtomentose when young and subglabrous in age, greyish orange (5B6), brownish orange to brownish yellow (5C4–5C8) at first, and gradually changing to light brown, yellowish brown to brown (5D5–5D8, 6D5–6D8) when mature. Context 8–10 mm thick at centre, white, unchanging when exposed to air. Tubes 4–6 mm deep, depressed or nearly free near the stipe in age, whitish, unchanging when cut. Pores 2–3 per mm, circular, white at first, becoming pale yellow (3A3–4A3) in age, unchanging when bruised. Stipe 60–70 × 20–25 mm, central, equal or slightly swollen downwards, brittle, stuffed with a soft pith, becoming hollow or developing several cavities in age, surface dry, coarsely tomentose to floccose-scaly, not reticulate, concolourous with pileus or paler, unchanging when bruised; basal mycelium white; stipe context white, unchanging when exposed. Odour none and taste mild.

Basidiospores (6.5)7–9(9.5) × 3.5–4 μm, Q = (1.77)1.8–2.5(2.57), Q_m_ = 2.01 ± 0.25, elliptic, cylindrical to somewhat oblong, smooth, thin-walled, yellowish to yellowish brown in 5% KOH and yellow brown to dark brown in Melzer’s reagent. Basidia 24–38 × 6–8 μm, 4-sterigmate, clavate, thin-wall, yellowish white to hyaline in 5% KOH. Pleurocystidia not observed. Cheilocystidia 37–100 × 6–10 μm, abundant and conspicuous, narrowly fusoid to cylindrical, smooth, thin-walled, hyaline. Hymenophoral trama subparallel, smooth or coarse, yellowish white to hyaline in 5% KOH, with hyphae 10–22 μm broad. Pileipellis a trichoderm, consisting of interwoven hyphae 7–10 μm in width, covered with yellowish brown to brown pigment on surface in 5% KOH, dark brown to rusty brown in Melzer’s reagent; terminal cells 30–95 × 7–10 μm, cylindrical or nearly clavate. Pileal trama subregular, composed of branched and interwoven hyphae up to 12–18 μm in width. Stipitipellis hyphae oriented in various directions, subparallel to repent, hyphae 6–15 μm broad, usually covered with yellowish brown to brown pigment in 5% KOH, end cells 30–90 × 6–15 μm, thin walled. Clamp connections present in all tissue.

Additional specimens examined: CHINA. Hainan Province, Ledong County, Jianfengling National Forest Park, at 18°44′N, 108°52′E, alt. 900 m, 4 July 2013, Ming Zhang (GDGM42986).

Habitat and distribution: Solitary or scattered on soil in mixed broadleaf-coniferous forests, mainly dominated by *Cyclobalanopsis* spp. and *Castanopsis* spp., alt. 900 m. Currently known from Hainan Province, China.

Notes: The combined morphological characters include the brownish orange to yellowish brown pileus covered with fibrillose or velvet-subtomentose when young and nearly smooth in age, the white context and tubes unchanging when bruised, the hollow and brittle stipe, elliptic to cylindrical basidiospores, and the longer cheilocystidia up to 100 μm; which allowed *G. pseudolongicystidiatus* to be easily separated from other species of the genus.

Phylogenetically, *G. pseudolongicystidiatus* is nested into the sect. *Longicystidiatus* and closely related to *G. longicystidiatus* and *G. paralongicystidiatus*. However, *G. longicystidiatus*, originally described from Japan, differs in the yellow-brown pileus, the presence of the pleurocystidia (38–140 × 12–21 μm), and the broader basidiospores with a smaller Q_m_ value (1.56 ± 0.24) (Nagasawa 2001; Xie et al. 2022); *G. paralongicystidiatus* Davoodian, recently reported from Costa Rica, differs in its brown to pinkish brown pileus covered with tomentose to finely matted or fine squamules or furfur, broader basidiospores (4.4)5.1–5.7(6.4) µm, and shorter cheilocystidia (17–55 × 6–13 µm)(Davoodian et al. 2018).

*Gyroporus* section *Pallidus* Ming Zhang & T.H. Li sect. nov.

Fungal Name: FN570999

Type species: *Gyroporus pallidus* Ming Zhang & T.H. Li

Etymology: “*Pallidus”* refers to the pale colour of basidiomata.

Basidiomata small to medium-sized. Pileus convex to applanate, dry, subtomentose, white, yellowish withe, yellowish brown, brownish orange to brown, without red or purple tinge; context white, unchanging when injured. Hymenophore poroid, white, unchanging when bruised. Stipe central, surface dry, glabrous or subtomentosus, concolourous with pileus or paler, unchanging when handled; basal mycelium white; annulus absent. Basidiospores ellipsoid to broadly ellipsoid, smooth, thin-walled, yellowish in 5% KOH. Basidia clavate, 4-spored, hyaline in 5% KOH. Cheilocystidia clavate to subfusiform, thin-walled, yellowish to hyaline in 5% KOH. Pleurocystidia not observed. Pileipellis a cutis or trichodermium, composed of interwoven, thin to thick-walled hyphae, colourless or yellowish in 5% KOH. Clamp connections present in all tissues.

Notes: The *G*. sect. *Pallidus* was well-supported as an independent branch in the phylogenetic trees (Figures 1 and 2), and four species, *G. alboluteus, G. memnonius, G. pallidus* and *G. subalbellus* were included. Of which, *G. alboluteus* and *G. pallidus* were newly discovered in the present study, *G. memnonius* was recently reported from southern China (Xie et al. 2022), and *G. subalbellus* originally reported in North America (Murrill 1910). In addition, two specimens named as “*G*. cf. *castaneus”*, and two unnamed sequences labelled as “OR182” and “BOS472” were also included in this section.

**Figure 8.**
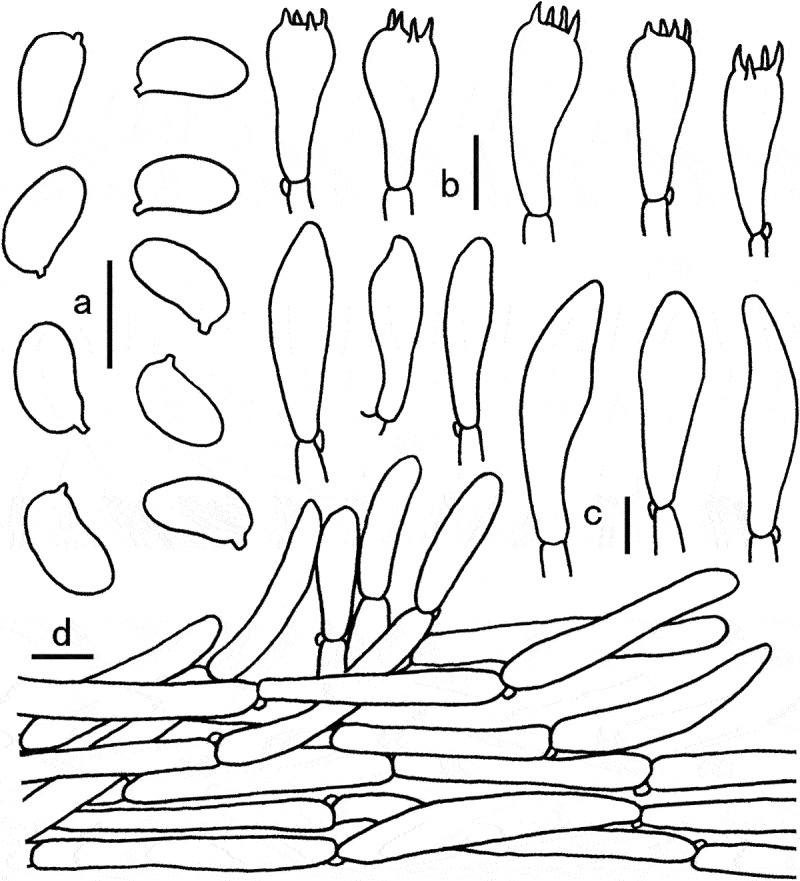
Microscopic features of *Gyroporus alboluteus*. a. Basidiospores; b. Cheilocystidia; c. Basidia; d. Pileipellis. Bras = 10 μm.

*Gyroporus alboluteus* Ming Zhang & T.H. Li sp. nov. Figure 6e–f, 8

Fungal Name: FN570979

Etymology: “*alboluteus*” refers to the yellowish white to pale yellow colour of the pileus.

Diagnosis: This species is characterised by its small basidiomata, pale yellow to pale orange pileus, white hymenophore and context unchanging when bruised, elliptical to cylindrical basidiospores (8)8.5–9.5(10) × 4.5–5 μm.

Holotype: CHINA. Guangdong Province, Shaoguan City, Shixing County, Chabaling National Natural Reserve, alt. 600 m, 15 July 2008, Tai-Hui Li (GDGM25474).

Basidiomata small. Pileus 2–3 cm broad, hemispheric, convex to plane, dry, fibrillose, velvet-subtomentose when young and nearly glabrous in age, white at first, pale yellow to pale orange at maturity, paler towards margin. Context 3–4 mm thick at centre, fleshy, white, unchanging when exposed. Tubes 3–4 mm deep, depressed or nearly free at stipe in age, white, unchanging when bruised. Pores 2–3 per mm, circular, white, unchanging when bruised. Stipe 30–50 × 6–10 mm, central, equal or slightly swollen downwards, brittle, stuffed with a soft pith, becoming hollow or developing several cavities in age, surface dry, glabrous or with white pruina, concolourous with pileus or paler, unchanging when handled, with white basal mycelium; stipe context white, unchanging when exposed. Odour none. Taste mild.

Basidiospores (8)8.5–9.5(10) × 4.5–5 μm, Q = (1.6)1.7–2(2.1), Q_m_ = 1.81 ± 0.13, elliptical, cylindrical to somewhat oblong, smooth, thin-walled, yellowish to yellowish brown in 5% KOH. Basidia 24–35 × 10–13 μm, 4-sterigmate, clavate, thin-wall, yellowish white to hyaline in 5% KOH. Pleurocystidia not observed. Cheilocystidia 28–40 × 8–15 μm, abundant and conspicuous, narrowly fusoid to cylindrical, smooth, thin-walled, hyaline. Hymenophoral trama subparallel, smooth or coarse, yellowish white to hyaline in 5% KOH, with hyphae 6–10 μm broad. Pileipellis a cutis, consisting of suberect to slightly interwoven hyphae 5–12 μm in width, covered with yellowish brown to brown pigment on surface in 5% KOH, terminal cells 30–95 × 7–10 μm, cylindrical or nearly clavate. Pileal trama subregular, composed of branch, parallel to slightly interwoven hyphae, 5–15 μm in width. Stipitipellis composed of thin-walled hyphae, 5–12 μm wide, light yellow in 5% KOH. Stipe trama composed of cylindrical, light yellow in 5% KOH, thin-walled, interwoven hyphae 5–12 μm wide. Clamp connections frequently present in all tissues.

Additional specimens examined: CHINA, Guangdong Province, Shaoguan City, Shixing County, Chebaling National Natural Reserve, alt. 640 m, 22 June 2014, Ming Zhang (GDGM86706).

Habitat and distribution: Solitary or scattered on soil in mixed forest dominated by Fagaceae trees, and mixed with *Pinus massoniana* Lamb. Currently known from Guangdong Province, China.

Notes: The combined morphological features of the small basidiomata, the pale yellow to pale orange coloured pileus, the white hymenophore and context unchanging when bruised, and the elliptical to cylindrical basidiospores make *G. alboluteus* easily distinguished from other species of *Gyroporus*. Ecologically, *G. alboluteus* is distributed in subtropical mixed forests, which are dominated by Fagaceae trees and mixed with a small amount of *Pinus massoniana.*

Phylogentically, *G. alboluteus* formed an independent clade in the *sect. Pallidus*, and was related to “*G*. cf. *castaneus”, G. memnonius, G. pallidus* and *G. subalbellus* Murrill, but they can be separated from each other by genetic distance. Besides, “*G*. cf. *castaneus”* distributed in northeastern China, differs in its larger basidimata (pileus can up to 10 cm broad), yellow to yellow-brown pileus, yellow-brown to orange-brown stipe, broader basidiospores [(4–)4.5–6(–7) µm], and trichodermium pileipellis (Xie et al. 2022). *Gyroporus memnonius*, recently described from southern China, differs in its larger basidiomata up to 6 cm broad, dark brown pileus, yellowish brown stipe, and slightly thick-walled basidiospores up to 0.5 µm thick (Xie et al. 2022). Another new species to be described in present paper, *G. pallidus*, differs in its larger basidiomata, reddish brown to dark brown pileus, and broader basidiospores [8–10 × 5–6 µm, Q = (1.3)1.45–1.81(1.9)]; additionally, *G. pallidus* was distributed in the southern margin of the temperate zone, and currently only known associated with *Castanea mollissima* BL. *Gyroporus subalbellus*, originally reported from North America, differs in its larger basidiomata (pileus up to 12 cm broad), apricot buff, pinkish buff to orange cinnamon pileus, and lager basidiospores measuring 8–14 × 4–6 µm (Murrill 1910; Bessette et al. 2000).

**Figure 9.**
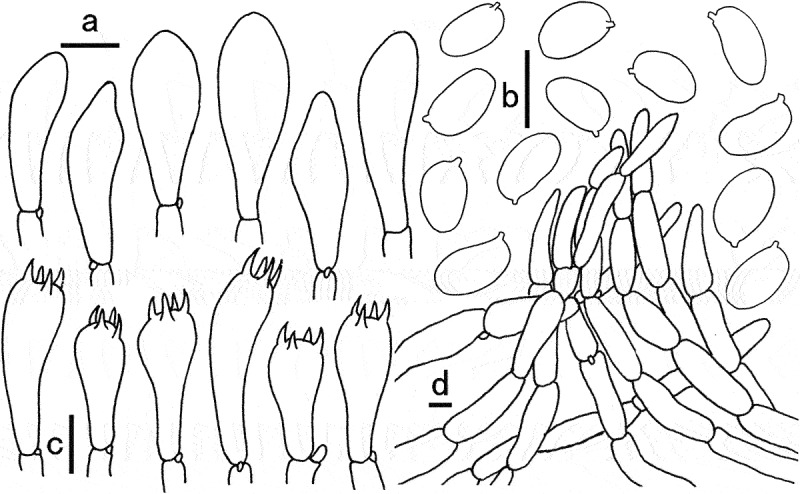
Microscopic features of *Gyroporus pallidus*. a. Cheilocystidia; b. Basidiospores; c. Basidia; d. Pileipellis. Bras = 10 μm.

*Gyroporus pallidus* Ming Zhang & T.H. Li sp. nov. Figure 6g–h, 9

Fungal Name: FN570982

Etymology: “*pallidus*” refers to the paler basidiomata colour to compare with *G. castaneus*.

Diagnosis: This species is characterised by its small basidiomata, brownish orange to light brown pileus usually cracked into small scales on the surface, white to yellowish white context unchanging when bruised, and elliptical basidiospores 8–10 × 5–6 µm.

Holotype: CHINA. Henan Province, Xinyang City, alt. 400 m, 22 July 2016, Ming Zhang (GDGM46387).

Basidiomata small to medium-sized. Pileus 3–5 cm broad, convex when young, then applanate with age; margin decurved at first, then slightly upward when old; surface dry, subtomentose, usually cracking into small scales when mature or in dry conditions, brownish orange, light brown to brown (5C4–5C6, 5D5–6D5); context 3–4 mm thick, white, unchanging in colour when injured. Hymenophore adnate to slightly depressed around stipe when mature, 3–5 mm long, white (1A1) when young, yellowish white (3A2) when mature, unchanging when bruised; pores angular to roundish, 2–3 per mm, white to yellowish white, unchanging when bruised. Stipe 4–6 × 0.6–1.2 cm, central, sub-cylindrical to clavate, concolourous with pileus, slightly paler to yellowish brown to yellowish downward the base; surface roughened, unchanging when bruised; context white to yellowish white, spongy when young and then hollow in age, unchanging when bruised. Odour none. Taste mild.

Basidiospores 8–10 × 5–6 µm, Q = (1.3)1.45–1.81(1.9), Q_m_ = 1.61 ± 0.15, smooth, elliptical, to somewhat broadly elliptical, yellowish in 5% KOH. Basidia 22–33 × 9–12 µm, clavate, 4-spored, hyaline in 5% KOH. Cheilocystidia 28–35 × 9–13 µm, clavate to subfusiform, thin-walled, yellowish white to hyaline in 5% KOH. Pleurocystidia not observed. Tube trama composed of 5–15 µm wide parallel hyphae, hyaline to yellowish in 5% KOH. Pileipellis a trichoderm, composed of thin-walled, elongated, and slightly interwoven hyphae, 7–22 µm wide, hyaline to yellowish in 5% KOH; terminal cells 23–100 × 8–22 µm, clavate to subcylindrical, with obtuse apex. Pileal trama made up of hyphae 5–25 μm diam, colourless in 5% KOH. Stipitipellis composed of thin-walled hyphae, 3–8 μm wide, light yellow in 5% KOH. Stipe trama composed of cylindrical, light yellow in 5% KOH, thin-walled, interwoven hyphae 8–23 μm wide. Clamp connections frequently present in all tissues.

Habitat and distribution: Solitary or scattered on soil under *Castanea mollissima* BL. in subtropical chestnut plantations. Currently only known from Henan Province, China.

Additional specimens examined: CHINA. Henan Province, Xinyang City, Renhua County, alt. 300 m, 22 July 2016, Ming Zhang (GDGM46275, GDGM46509, GDGM46401, GDGM46405); same location, alt. 350 m, 22 July 2016, Xiang-Rong Zhong & Tai-Hui Li (GDGM46419, GDGM46433).

Notes: Phylogenetic analyses shown that the specimens of *G. pallidus* formed a well supported lineage and nested into the sect. *Pallidus. Gyroporus alboluteus* and *G. memnonius* are two closely related species in phylogeny. Indeed, *G. memnonius* resembles *G. pallidus* in morphology, but differs in its stronger basidiomata, narrower basidiospores (4–5 μm wide) and smaller basidia 19–26 × 6 μm (Xie et al. 2022). *G. alboluteus* can be easily distinguished by its pileus colour (see above in *G. alboluteus*). Besides, two specimens from China named as “*G*. cf. *castaneus*” also closely related to *G. pallidus*. However, the former differs in its lager basidiomata, narrower basidiospores [(4–)4.5–6(–7) μm] with a large Q_m_ value (1.81 ± 0.16), and distributed in the temperate regions of northeastern China (Xie et al. 2022).

Morphologically, *G. pallidus* resembles *G. castaneus, G. paramjitii, G. punctatus* Lj.N. Vassiljeva and *G. tuberculatosporus*. However, *G. castaneus*, originally reported from Europe, differs in its larger basidiomata (pileus can up to 10 cm broad) and basidiospores [8–12(14) × 4.5–6(7) μm] (Heinemann and Rammeloo 1979; Moser 1983; Castro and Freire 1995); *Gyroporus paramjitii*, originally described from India, differs in its dark brown to red brown basidiomata, smaller basidia (11–16 × 6 μm), and larger basidiospores (7.5) 8–11.6 (13) × 5–6.6 (7) μm (Das et al. 2017; Xie et al. 2022); *Gyroporus punctatus*, originally described from the south of Russian Far East, differs in its rugulose to reticulate pileus, rugulose stipe, and larger basidiospores (up to 12 μm long) (Vassiljeva 1950; Nagasawa 2001); *G. tuberculatosporus*, originally reported from southwestern China, differs in its larger and yellowish brown basidiomata, large and broad basidiospores (9–11.3 × 5–8.7 μm) (Zang 2006).

Two new species *G. porphyreus* and *G. subglobosus* recently reported from China, also similar to *G. pallidus*. However, *G. porphyreus* differs in its yellow-brown, redbrown to purple pileus, brown to red-brown stipe, and narrower basidiospores (4–5.5 μm wide) with a relatively large Q value (1.4–2.56) (Xie et al. 2022); *G. subglobosus* differs in its yellowish brown, red-brown to dark brown pileus, brown to red-brown stipe, and subglobose basidiospores (6.5–9.5 × 5–7 μm) with a small Q value (1.1–1.5), besides, *G. subglobosus* can naturally distributed in northeastern China, and associated with *Pinus koraiensis* Siebold et Zuccarini, *Quercus mongolica* Fischer ex Ledebour, or *Castanopsis kawakamii* Hayata.(Xie et al. 2022). Additionally, *G. porphyreus* and *G. subglobosus* nested into the sect. *Castaneus*, and can be easily distinguished from *G. pallidus* by the lager genetic distances.

## Discussion

In this study, a phylogenetic overview of the genus *Gyroporus* was carried out on the basis of the combined sequences ITS-nrLSU and *atp6* datasets, four new sections within the genus were proposed, i.e. *G*. sect. *Castaneus, G*. sect. *Cyanescens, G*. sect. *Longicystidiatus* and *G*. sect. *Pallidus*, and five new species, *G. alboluteus, G. atrocyanescens, G. pseudolongicystidiatus, G. pallidus* and *G. subcaerulescens*, were discovered from China.

In sect. *Castaneus, G. castaneus* has been widely reported in Europe, North America and eastern Asia. However, the reported collections of “*G. castaneus*” are actually a complex consisting of several different taxa (Das et al. 2017; Davoodian et al. 2018; Xie et al. 2022); for example, the specimens labelled as “*G. castaneus*” represent obviously more than one species in the phylogenetic tree (Figure 1). The specimens of this complex from subtropical and tropical regions of Asia or other continents represent different species, such as *G. mcnabbii, G. memnonius, G. naranjus, G. paramjitii, G. pallidus* etc. It is believed that further studies with more samples will contribute more to reveal the diversity of *G. castaneus* complex.

The sect. *Cyanescens* formed a monophyletic group in the phylogenetic tree (Figures 1 and 2), and consisted of species with cyanescent oxidation reactions. *Gyroporus atrocyanescens* and *G. subcaerulescens* are well nested into this section, and closely related to the Chinese species *G. flavocyanescens*, but they can be separated by the morphological features and the genetic distance. In this section, the species from Southern Hemisphere clustered together, while the species from Northern Hemisphere formed two well supported clades in the *atp6* phylogenetic tree (Figure 2), which was consistent with the previous study by Davoodian et al. (2018). *Gyroporus cyanescens* was reported to be widely distributed in China in the past (Bi and Zheng 1990, 1994; Ying and Zang 1994; Mao 2000; Li et al. 2015), and a recently study proved that the distribution of *G. cyanescens* in China is highly suspectable and specimens fully identical to the European species has not been found yet (Huang et al. 2021). Specimens from temperate regions of China labelled as “*G. cyanescens*” could be *G. alpinus*, and specimens from subtropical or tropical regions of China with obvious cyanescent oxidation reactions could be *G. brunneofloccosus, G. flavocyanescens* or *G. atrocyanescens*.

The sect. *Longicystidiatus* was well supported (Figures 1 and 2; BS/BPP = 99%/1; BS/BPP = 100%/1), and formed a sister relationship with the sect. *Cyanescens*. However, species in this section lack of cyanescent oxidation reaction and can be easily distinguished from other species in *Gyroporus* by their very large and conspicuous cystidia, especially in *G. longicystidiatus* and *G. pseudolongicystidiatus*, the size of cheilocystidia can up to 100 µm in length.

The sect. *Pallidus* formed a monophyletic clade in the phylogenetic trees (Figures 1 and 2), and four species were included. Species in this section are difficult to distinguish from the species in sect. *Castaneus* in morphology, but they can be easily separated from each other in phylogeny. Besides, species in sect. *Pallidus* usually have paler pileus colour, and without red or purple tinge to compare with species in the sect. *Castaneus*. The species “*G*. cf. *castaneus*”, described from China in Xie et al. (2022), has been proved to belong to the sect. *Pallidus*, and represents a different species from *G. castaneus.*

As noted in previous studies, species of *Gyroporus* are widely distributed in China and eastern Asia. Although several new species have been reported, there are still a larger number of unidentified specimens waiting to be studied, and numerous additional hidden species would be revealed based on more collections and DNA molecular evidences in the future.

**Key to sections and species of *Gyroporus* from China**
**1**. Basidiomata not cyanescent …. … . … . … . … . … . **2**
  **1**. Basidiomata cyanescent … … . … . … . … . … . **4 (sect. *Cyanescens*)**
**2**. Cheilocystidia shorter, usually less than 50 μm long … . … . … . … . … . … . … . … . … . … . … . … . … . … . … . … . … . … . … . … . … . … . … . … . … . **3**
  **2**. Cheilocystidia longer, some much longer than 50 μm, up to 100 μm long … . … . … . … . … . … . **8 (sect. *Longicystidiatus*)**
**3**. Pileus without red or purple tinge, white, brown, yellowish brown to dark brown, stipe concolourous with pileus or paler; pileipellis as a cutis or trichoderm … . … . … . … . … . **9 (sect. *Pallidus*)**
  **3**. Pileus always with more or less red or purple tinge, yellow-brown, orange-brown, brown to red brown, stipe concolourous with pileus or darker, pileipellis trichodermium … … **12 (sect. *Castaneus*)**
**Section *Cyanescens***
**4**. Only hymenophore and pileus context slowly changing to pastel blue when bruised … . … . … . … . … . … . … . … . … . … . … . … . … . … . … . … . … . … . … . … . … . … . … . … . … . … . … . … … ***G. subcaerulescens***
  **4**. All parts of basidiomata changing to blue when bruised … . … . … . … . … . … . … . … . … . … . … . … . … . … . … … . **5**
**5**. Pileus and stipe obviously brown, from brownish orange, light brown, light reddish brown to dark brown, with brown floccose-scales and long hairs or villi; context white, turning light turquoise at first, then quickly becoming dark turquise or dark blue when exposed; basidiospores 8.5–10 × 5–6 µm … . … . … . … . … . … . … . … . … . … . … . … . … . … . … . … . … . … . … . … . … . … . … … . ***G. brunneofloccosus***
  **5**. Pileus and stipe paler than above, with little or without brown tinge, from white, ivory yellow, greyish-yellow, olive yellow, flavous, grey-yellow to grey-orang; brown floccose-scales and long hairs abscent or much less obvious … … . … . … . … . … . … . … . … . … . … . … . … . … . … . … . … . … . … . … . … . … . … . … . … . **6**
**6**. Basidiomata immediately and intensely turning dark blue to deep blue when bruised; pileus white to greyish yellow, covered with greyish yellow floccose squamules; basidiospores 7.5–10 × 4.8–6 μm … . … . … . … . … . … . … . … . … . … . … . … . … . … . … . … . … . … . … . … . … . … . … . … . … . … . … . ***G. atrocyanescens***
  **6**. Basidiomata gradually becoming blue to dull blue when bruised … . … . … . … . … . … . … . … . … . … . … . … . **7**
**7**. Pileus ivory yellow to grey-orange or brownish-yellow, covered with scaly to floccose squamules; basidiospores 7–10 × 5.5–7.5 µm; basidia long and slender, 35–55 × 7–12 µm; and distributed in alpine forests … . … . … . … . … . … . … . … . … . … . … . … . … . … . … . … . … . … . … . … . … . … . … . … . ***G. alpinus***
  **7**. Pileus flavous, dull yellow, grey-orange to greyish-orange, nearly glabrous or somewhat fibrillose to finely tomentose; basidiospores 8–10 × 5.5–6.5 µm; and distributed in tropical forests dominated by Fagaceae trees … . … . … . … . … . … . … . … . … . … . … . … . … . … . … . … . … . … . … . … . ***G. flavocyanescens***
**Section *Longicystidiatus***
**8**. Cheilocystidia broader, 23–98 × 9–21 μm; pileus 2.7–9 cm broad, subtomentose, drab, yellow-brown to dark yellow-brown; stipe light yellowish brown to brown; basidiospores 7–9 × 3.5–6 μm … . … . … . … . … . … . … . … . … . … . … . … . … . … . … . … . … . … . … . … . … . … . … . … . … . ***G. longicystidiatus***
  **8**. Cheilocystidia narrower, 37–100 × 6–10 μm; pileus 5–10 cm broad, subglabrous when mature, brownish orange to brownish yellow; basidiospores 8–10 × 5.5–6.5 µm … . … . … . … . … . … . … . … . … . … . … . … . … . … . … . … . … . … . … . … . … . … . … . … . … … ***G. pseudolongicystidiatus***
**Section *Pallidus***
**9**. Pileus white, pale yellow to pale orange, fibrillose or velvet-subtomentose when young and nearly glabrous in age; stipe surface glabrous or with white pruina; basidiospores 8.5–9.5 × 4.5–5 μm; pileipellis a cutis, composed of suberect, interwoven hyphae covered with yellowish brown to brown pigment on surface in 5% KOH; terminal cells 30–95 × 7–10 μm … . … . … . … . … . ***G. alboluteus***
  **9**. Pileus never white to pale yellow or pale, without yellowish tinge … . … . … . … . … . … . … . … . … . … … **2**
**10**. Basidiomata larger, pileus up to 10 cm broad, yellow to yellow-brown; basidiospores 8–10 × 4.5–6 μm; distributed in temperate areas … . … . … . … . … . … . … . … . … . … . … . … . … . … . … . … … . ***G*. cf. *castaneus***
  **10**. Basidiomata smaller, pileus less than 10 cm broad … . … . … . … . … . … . … . … . … . … . … . … . … . … . … . … . … . **11**
**11**. Pileus 4.8–6 cm broad, dark brown; stipe subtomentose, brown-yellow to yellowbrown; basidiospores 8–10 × 4–5 μm, slightly thick-walled; pleurocystidia 17–40 × 4–10 μm, subfusiform or fusiform; pileipellis a trichoderm, composed of thin to slightly thick-walled hyphae, light yellow in KOH; terminal cells 38–105 × 9–15 μm … . … . … . … . … . … . … . … . … . … . … . … . … … ***G. memnonius***
  **11**. Pileus 3–5 cm broad, usually cracking into small scales when mature or in dry conditions, brownish orange, light brown to brown; stipe concolourous with pileus, slightly paler to yellowish brown to yellowish downward the base; basidiospores 8–10 × 5–6 µm; pileipellis a trichodermium, composed of thin-walled, elongated, and slightly interwoven hyphae, hyaline to yellowish in 5% KOH; terminal cells 23–100 × 8–22 µm … . … . … . … . … . … . … . … . … . … . … . … . ***G. pallidus***
**Section *Castaneus***
**12**. Basidiomata larger, pileus up to 10 cm broad, yellowish brown to reddish brown; basidiospores 9–11.3 × 5–8.7 μm; distributed in tropical forests … . … . … . … . … … ***G. tuberculatosporus***
  **12**. Basidiomata smaller, pileus less than 10 cm broad … . … . … . … . … . … . … . … . … . … . … . … . … . … . … . … . … . **13**
**13**. Basidiospores subglobose to ellipsoid (Q_m_ < 1.5) … … . … . … . … . … . … . … . … . … . … . … . ***G. subglobosus***
  **13**. Basidiospores oval to ellipsoid (Q_m_ > 1.5) … … . … . … . … . … . … . … . … . … . … . … . … . … . … . … . … . … . … . … . **14**
**14**. Pileus yellow-brown, brown to red-brown when young, then purple; stipe brown, pale to red-brown; basidiospores 7–10.5 × 4–5.5 μm … . … . … . … . … . … . … . … . … . … . … . … . … . … . … … ***G. porphyreus***
  **14**. Pileus red-brown, orange brown to dark red-brown; stipe red-brown to dark red-brown; basidiospores 7–9 × 5–6 μm … . … . … . … . … . … . … . … . … . … . … . … . … . … . … . … . … . … . … . … . … . … . … . ***G. paramjitii***
